# The acute mania of King George III: A computational linguistic analysis

**DOI:** 10.1371/journal.pone.0171626

**Published:** 2017-03-22

**Authors:** Vassiliki Rentoumi, Timothy Peters, Jonathan Conlin, Peter Garrard

**Affiliations:** 1 Neuroscience Research Centre, Molecular and Clinical Science Research Institute, St. George’s, University of London (SGUL), London, United Kingdom; 2 Institute of Archaeology and Antiquity, University of Birmingham, Birmingham, United Kingdom; 3 School of Humanities, University of Southampton, Southampton, United Kingdom; The University of Newcastle, Australia, AUSTRALIA

## Abstract

We used a computational linguistic approach, exploiting machine learning techniques, to examine the letters written by King George III during mentally healthy and apparently mentally ill periods of his life. The aims of the study were: first, to establish the existence of alterations in the King’s written language at the onset of his first manic episode; and secondly to identify salient sources of variation contributing to the changes. Effects on language were sought in two control conditions (politically stressful vs. politically tranquil periods and seasonal variation). We found clear differences in the letter corpus, across a range of different features, in association with the onset of mental derangement, which were driven by a combination of linguistic and information theory features that appeared to be specific to the contrast between acute mania and mental stability. The paucity of existing data relevant to changes in written language in the presence of acute mania suggests that lexical, syntactic and stylometric descriptions of written discourse produced by a cohort of patients with a diagnosis of acute mania will be necessary to support the diagnosis independently and to look for other periods of mental illness of the course of the King’s life, and in other historically significant figures with similarly large archives of handwritten documents.

## 1. Introduction

It is well known that, in the course of his 60 year reign as King of Great Britain and Ireland (1760–1820), George III suffered from recurrent episodes of physical and mental ill health [1]. These illnesses compounded many of the great political issues of the day, and gave rise to at least one major constitutional crisis—the Regency Bill, which was debated during the King’s first documented period of mental derangement (1787–88). Towards the end of his life King George became cognitively impaired, and authority was transferred to his eldest son, who acted as Regent between 1811 and his accession as George IV in 1820.

The cause of the King’s psychiatric episodes and cognitive impairment has been the subject of much speculation. At the time, the King’s behaviour (agitation, rambling incoherent speech, and episodes of violence and sexual impropriety) were treated by restraint (a method favoured by specialists in ‘insanity’). With the emergence of psychiatry as a medical discipline in the late nineteenth century the episodes were reinterpreted as manifestations of manic-depressive psychosis [2]. In 1966 Macalpine and Hunter proposed, instead, that the King’s illnesses were due to recurrent attacks of acute porphyria, and that this inherited metabolic disorder, characterized by paroxysms of physical and mental disturbance, could be identified in succeeding generations and traced as far back in the King’s ancestry as Mary Queen of Scots (1542–87) [3, 4].

Despite early skepticism from some quarters [5] the porphyria hypothesis gained widespread acceptance, not only in the medical sphere [6], but among historians [1, 7] and in popular culture [8]. Recent evaluations of the King’s medical records by Peters and colleagues [9, 10], however, revealed not only that the evidence for porphyria is insubstantial, but that its interpretation was seriously flawed. In the present paper we interrogate a contemporary source material, in the form of written language production in the King’s letters. These have been preserved in manuscript form in several historical archives, and in many cases transcribed and annotated for print publication.

Advances in the automatic statistical analysis of textual material have produced methods by which digitized samples of written text can be rapidly and reliably categorized. Analyses of digitized samples of spoken language using automated, computer-based methods in schizophrenia [11] have demonstrated the impact of disordered thinking on language use, and a recent study [12] indicated significant differences between the language production of bipolar disorder patients compared to their control counterparts. Computational approaches that exploit machine learning techniques have been successfully deployed to distinguish the language used by patients suffering from different types of dementia [13], while computer-based stylometric analyses have been used to examine the written output of literary figures, finding evidence of declining intellectual function in individuals with degenerative dementia years before symptoms first appeared [14].

In the field of literary scholarship, the techniques of computer-based stylometry and automatic authorship attribution pioneered by Burrows [15] have demonstrated measurable differences between individual authors, texts originating from the 18th and 19th centuries, and groups of male and female writers. In spite of the survival of large quantities of *written* language output from the 17^th^ century onwards these techniques have not previously been extended to the study of historical figures. Written correspondence between King George and senior parliamentarians survives in manuscript form and transcripts of the majority have been published [16], the content for the most part suggesting an active engagement with political events. We hypothesised that it would be possible to identify and characterise changes in written language coinciding with known periods of mental disturbance using computer-based language analysis techniques.

To test this hypothesis we undertook a series of analyses of subsets of letter texts, with a view to addressing the following questions:

1. Is the King’s language at the onset of the manic episode measurably different from that immediately preceding and/or following it? What sort of differences can one detect during this phase, and are they in any way consonant with the language abnormalities that are described in acute mania?
2. Is the change an effect of disease, or does it reflect non-pathological influences, such as i) the stress of leading a powerful nation through turbulent times, or even nothing more than ii) seasonal variation in language use?
3. Are the intra-individual language characteristics across specific intervals of King George’s lifetime stable? For instance, does a stressed or manic period, when examined separately, exhibit relative language stability, which could be indicative of a specific language pattern occurring in each period? Demonstration of clear language distinctions between the alleged manic phase and other periods, in combination with the existence of language homogeneity within each period in isolation would be consistent with the notion that the king’s psychological pathology was indeed reflected in his language behaviour.

The current study addresses these questions by applying a computational approach based on machine learning (text classification and feature selection) to a text corpus consisting of King George III’s handwritten letters. We set out to obtain measures derived from textual characteristics known to be sensitive to mental illness [12] and to plot their changes over time.

We focus on the King’s first manic episode of 1788–1789, which is said to have been the most severe [17, 18], aiming: i) to confirm the presence of differences between letters written during this period and those from a number of control conditions across a range of linguistic variables; ii) to identify the most informative sources of variation between letters from the different periods of interest; and iii) to describe any patterns and similarities intrinsic to the manic period. We address these aims using computational approaches to the estimation of syntactic complexity and lexical variety, and information theory characteristics in digitized texts of the king’s letters.

## 2. Materials and methods

### 2.1 Data sets

Between accession in 1760 and the beginning of his late life decline in 1810, King George III was an assiduous letter writer. The majority of his letters to and from his various correspondents have since been transcribed and published [16, 19]. All the letters prior to 1806 (when visual deterioration forced him to use an amanuensis) were written in his own hand, and the details of where he wrote the letter, the date and even the time of writing to the nearest minute, were also usually recorded. Nearly all letters were personally signed with a full signature, though some were just initialed. Only a few letters (mostly those written to close associates) were left unsigned. Having examined some 500 of his letters dating to the period between 1750 and 1810, we can confirm that all of these were written by King George himself, and that the published transcriptions are accurate and not significantly edited, other than with some minor and occasional editorial corrections of dates [20]. Thus, we can confidently state that the letters used in this study have not been subjected to significant assistance or editorial alterations.

We conducted experiments on ten data sets (presented in Table 1) consisting of letters written by the king to senior political figures, from seven different periods of his life. (We excluded letters not dealing with affairs of state—such as those written to family members—in the interests of maintaining thematic consistency.) Table 1 provides details for the structure of each of the data sets, including the dates between which the letters were written, the average number of words used in each letter subset and the published sources.

**Table 1:** Structure and characteristics of the data sets used in contrasts A to F.

Comparison		Date ranges of letters	Number of letters in subset	Average word tokens per letter	Relevant contrast	Expected outcome of ML classification	Data sources
A	Pre-mania	April 1788–September 1788	21	129.1	Mania related language change (immediate)	Distinguishable	Aspinal Vol. 1 [19] Stanhope Vol. 2 [55]
	vs.						
	Acute mania	October 1788–April 1789	31	152.4			
B	Acute mania	October 1788–April 1789	31	152.4	Mania related language change (immediate)	Distinguishable	Aspinal Vol. 1 [19] Stanhope Vol. 2 [55] Dobree [21]
	vs.						
	Post-mania	May 1788–October 1789	37	73.3			
C	Acute mania	October 1788–April 1789	31	152.4	Mania related language change (delayed)	Distinguishable	Aspinal Vol. 1 [19] Stanhope Vol. 2 [55] Fortescue [16]
	vs.						
	Mentally healthy, no stressors	October 1770–April 1771	47	85.1			
D	Acute mania	October 1788–April 1789	31	152.4	Stress related language change	Distinguishable	Aspinal Vol. 1 [19] Stanhope Vol. 2 [55] Fortescue [16]
	vs.						
	Mentally healthy, political stressors	April 1780–October 1781	42	127			
E	Mentally healthy: Spring & Summer	April 1770–September 1770	20	57.2	Seasonally-determined language change	Indistinguishable	Fortescue [16]
	vs.						
	Mentally Healthy: Autumn & Winter	October 1770–April 1771	47	85.1			
F	Mentally healthy: Autumn & Winter	October 1770–April 1771	47	85.1	Seasonally-determined language change	Indistinguishable	Fortescue [16]
	vs.						
	Mentally healthy: Spring & Summer	May 1771–October 1771	11	101			

Each data set embodies a comparison between intervals of interest, and incorporates published letters dating from the periods under investigation [16, 19, 21]. The comparisons made between these datasets address the research questions outlined above.

#### 2.1.1 Differences associated with manic period

In order to confirm the differences and specify the sources of variation in King George's written language during the onset of his manic episode compared to the periods that immediately preceded or followed it, the following comparisons were performed: A) Acute Mania vs Pre-Mania: letters written during the onset of the first manic episode between October 1788 and April 1789 [17] (Acute Mania set) were compared to those written during the six months before the beginning of the manic episode (Pre-Mania set); and B) Acute Mania vs Post-Mania: The Acute Mania set was compared to letters written during the six months after the manic episode (the Post-Mania set).

#### 2.1.2 Differences associated with stressful circumstances

To address the question of whether differences between letters written during and before/after the manic phase reflected a psychological response to stressful current events rather than a psychiatric illness, the Acute Mania set was compared to letters dating from 18 years earlier (October 1770 to April 1771) and 8 years earlier (October 1780 to April 1781).

The 1770–1771 period was politically stressful for King George owing to the Falklands Crisis, in which seizure by the Spanish of a British settlement on the islands was averted by the aggressive mobilization of British naval power [22]. The period between 1780 and 1781 was selected as a politically stressful time because of the culmination of the American war of Independence and subsequent resignation of Lord North as Prime Minister [23].

By contrast, the period between October 1788 and April 1789 as well as the six months preceding and following it did not coincide with any events that could be seen as politically difficult for King George [1]. It should be noted that: i) in both cases the dates spanned intervals identical to the period of the manic episode; and ii) the (politically stressful) Regency crisis of 1789 occurred during the period of mental illness but is considered to be a consequence, rather than a cause of it.

Data from these two periods allowed the following comparisons to be performed: C) Acute Mania vs 1770–1771; and D) Acute Mania vs 1780–1781.

#### 2.1.3 Seasonal differences

Letters written during an apparently healthy period of the king’s life (October 1770 to April 1771) were compared with those written six months before and immediately after this interval. These document set were chosen to target the question of whether language differences between the 1788–89 manic period and the preceding and subsequent six month periods merely reflected seasonal variation. The relevant comparisons were: E) October 1770 –April 1771 vs April 1770 –September 1770, and F) October 1770 –April 1771 vs May 1771 –October 1771.

#### 2.1.4 Stability of language characteristics within healthy and manic periods

To address the question of whether the characteristics of language used during the manic and healthy periods were stable, we assessed similarities among letters written during the period of acute mania and among those written during a healthy period (October 1770 to April 1771). Letters from the acute mania phase were subdivided into three equally sized, sequential subsets. Data from the first and last subsets were compared with those from the middle period. Those written between October 1770 and April 1771 were similarly subdivided and compared.

## 3. Data analysis

### 3.1 Analytical approach

The overall aim of the analysis was to identify a feature set that could correctly classify every text into a period of interest within the reign of King George III. This involved three consecutive analytical stages, the output of each providing input for its successor. In the first stage (feature extraction) values associated with 29 features were obtained from each text. Features, which are listed in detail in Table 2 and further explained in section 3.2, fell into two broad categories: i) features related to syntactic complexity; and ii) textual features. The latter included indices of lexical variation [24, 25], and measures derived from information theory. Features were obtained using Lu’s L2 Syntactic and Lexical Complexity Analyzers [26, 27], and Keyplex (a textual analysis program that provides values for a range of statistical and lexical measures). In addition, a compression ratio feature was computed using the zlib library of the Python programming language.

**Table 2:** Definitions of the syntactic and textual features used in the analysis.

Feature category	Feature		Abbreviation	Definition
*Syntactic complexity*	1	Mean length of clause	MLC	Number of words / number of clauses^1^
	2	Mean length of sentence	MLS	Number of words / number of sentences^2^
	3	Mean length of T-Unit	MLT	Number of words / number of T-units^3^
	4	Sentence complexity ratio	C/S	Number of clauses/number of sentences
	5	T-Unit complexity ratio	C/T	Number of clauses/number of T-units
	6	Complex T-Unit ratio	CT/T	Number of complex T-units^4^ / number of T-units
	7	Dependent clause ratio	DC/C	Number of dependent clauses^5^/ number of clauses
	8	Dependent clauses per T-Unit	DC/T	Number of dependent clauses / number of T-units
	9	Coordinate phrases per clause	CP/C	Number of coordinate phrases^6^/ number of clauses
	10	Coordinate phrases per T-Unit	CP/T	Number of coordinate phrases / number of T-units
	11	Sentence coordination ratio	T/S	Number of T-units / number of sentences
	12	Complex nominals per clause	CN/C	Number of complex nominals^7^ / number of clauses
	13	Complex nominals per T-Unit	CN/T	Number of complex nominals / number of T-Units
	14	Verb phrases per T-Unit	VP/T	Number of verb phrases / number of T-units
*Textual*	15	Lexical word variation	LV	Number of lexical word types / number of lexical words
	16	Bilogarithmic type token ratio	LogTTR	Log_10_ word types / Log_10_ total words (tokens)
	17	Noun variation	NV	Number of noun types / number of lexical words
	18	Adjective variation	ADJV	Number of adjective types / number of lexical words
	19	Modifier variation	MODV	Number of adjective types + adverb types / number of lexical words
	20	Adverb variation	ADVV	Number of adverb types / number of lexical words
	21	Corrected verb variation	CVV	Number of lexical verb types / √(number of verbs x 2)
	22	Verb variation	VV	Number of lexical verb types / number of lexical words
	23	Brunet's index W	W	N^v-0.165^ (N = number of word tokens; V = vocabulary)
	24	Hapax legomena	HL	Mean number of hapax legomena (once-used tokens) per 10 word block
	25	Pair hapax legomena	PHL	Mean number of pair hapax legomena (once-used token pairs) per 10 word block
	26	Simpson's diversity index	D	
	27	Dis legomena over vocabulary	DL/V	Mean number of dis legomenta (twice-used tokens) / number of word types
	28	Shannon entropy	H	
	29	Compression ratio	CR	Compressed (zipped) file size / uncompressed size

^1^ Clause = a structure consisting of at least a subject and a finite verb

^2^ Sentence = a group of words delimited with a punctuation mark that signals a sentence-ending

^3^ T-Unit = a main clause plus attached or embedded subordinate clause or other structure

^4^ Complex T-Unit = a T-Unit that contains a dependent clause

^5^ Dependent clause = a clause that cannot form a sentence on its own

^6^ Coordinate phrase = a phrase immediately before a coordinating conjunction (e.g. 'and', 'but', 'or')

^7^ Complex nominal = a sequence of words denoting a single concept

The second stage was feature selection, the aim of which was to identify features that could best distinguish among letters belonging to different periods of interest; the selected features also provide more fine-grained information concerning differences in language between periods of interest.

In the final stage—machine learning (ML) classification—the selected features were used to ‘train’ a classifier to assign groups of letters to one of the two periods under comparison. It was assumed that accurate ML classification would depend on the presence of systematic differences in language use between the periods under comparison. The computational basis of feature selection and ML classification are described briefly in sections 3.3 and 3.4 respectively, and presented in more detail in Section B in S1 File and Section C in S1 File. Feature selection and ML classification were implemented using the Waikato Environment for Knowledge Analysis (WEKA) [23] http://www.cs.waikato.ac.nz/ml/weka

### 3.2 Feature extraction

The complete set of 29 features that were extracted from all texts in the corpus are listed in Table 2, where they are subdivided into those relevant to syntactic complexity and those quantifying textual characteristics.

#### 3.2.1 Syntactic complexity features (1–14 in Table 2)

According to Ortega [28] syntactic complexity may be quantified in terms of the number of immediate constituents of a syntactic construction, which indicates how varied and sophisticated the production units or the grammatical structures are in writing, and has previously been used in analyses of narrative speech transcripts originating from patients with different subtypes of primary progressive aphasia [29]. Measures that characterize syntactic complexity include: the length of the production units (e.g. clauses, sentences or T-units); the amount of embedding or subordination; the amount of coordination; the range of surface syntactic structures; and the degree of sophistication of the syntactic structures employed [30].

#### 3.2.2 Textual features (15–29 in Table 2)

In the textual category, features 15–27 (up to and including Simpson’s diversity index [31] and Dis legomena over vocabulary) are relevant to lexical complexity and variation, while 28 and 29 are information theoretic measures. A subset of textual features (indicated in Table 2) are sensitive to document length, and we computed mean values for these features using sequential segments of 10 words within each letter. Lexical variation features include measures of the vocabulary range in each letter set, as estimated using the (mean segmental) bilogarithmic type/token ratio (MS Log TTR), higher values of which indicate greater lexical variation and lexical word variation (LV)—the ratio of the number of lexical word types to the total number of lexical words in a text [32]. Features 17–22 are more fine-grained variants of LV, sharing the same denominator (the number of lexical words), but using counts of nouns, verbs, adverb, adjectives and modifier types as numerators [33].

A related index is Brunet’s W, lower values of which imply a higher number of distinct word types, and thus a richer vocabulary (see Table 2 for a formal definition). Simpson’s D [31], gives the probability that two words that have been randomly selected from the text are the same, and thus quantifies the rate of word repetition in samples (lower values indicating less repetition). The dis legomena over vocabulary is derived by dividing the number of words that occur more than once by the number of word types, higher values implying a poorer vocabulary. Higher values of pair-hapax legomena (i.e. once used token-pairs), on the other hand, imply a more varied language use.

Information theory features [34, 35] are included, along with lexical variation features, in the textual category because language studies have shown strong inter-correlations among all these kinds of features: increased lexical richness is associated with increased entropy, and thus with a greater degree of randomness and uncertainty, which would be expected of a text with less repetitiveness and lower compressibility [36–38].

Of the two information theory features the first (feature 28) is the value of Shannon entropy (H) [34] computed in letters. In information theory, H is equivalent to the amount of information (measured in bits) that is added when the value of a previously unknown variable is obtained. The entropy of a random variable equates to its unpredictability. Shannon showed how information content [35] could be measured in written language, by empirically determining the accuracy with which a reader could predict the identity of sequentially revealed characters (including spaces) in a segment of text. Thus, H is related to the information content of language insofar as a less patterned sequence (e.g. sentence) that has a high value of entropy, is also highly informative, while a more patterned sentence would have low entropy and be less informative. In essence, Shannon Entropy quantifies the degree of randomness and uncertainty exhibited in the letters.

The second information theory feature is compression ratio (CR) [36], which quantifies the amount of repetitiveness in the language. Compression is achieved by condensing a piece of data such that it takes up less space but still contains the same amount of information [37]. Compression on texts works by omitting the words that are repeated, reducing the redundancy of the text. It follows that a repetitive text can be compressed to a greater extent than a non-repetitive one. CR is the ratio of the size of the compressed text to its uncompressed size. Entropy and compression ratio are thus tightly correlated: intuitively we expect a text with low entropy to have a high compression capacity, since its structure will be more patterned and predictable, while a text that carries a lot of new information will have high entropy and less potential for compression.

### 3.3 Feature selection

Not all features are of equal relevance in a classification problem, and identification of those making the largest contribution can improve classification performance, as well as providing insights into differences between samples. To establish the relevance of the features described above, we used the Information Gain (IG) algorithm for feature selection [39]. The selection of IG was based on its appropriateness for text domain related tasks [40] and for problems involving large numbers of features. IG is also faster than other feature selection methods such as wrappers [40].

IG works by identifying the features that are highly correlated with one or other of the classes (here, periods of letters). In other words, rather than using conventional statistical tests to determine if two groups of feature values are significantly different, IG investigates the usefulness of each feature in terms of its predictive value for the class in question (i.e. how much information each feature carries). A more formal explanation and definition of IG can be found in Section B in S1 File. The algorithm creates a decision tree to establish the purity of the groups into which each feature divides the to-be-classified instances. The IG associated with a feature is defined as the difference in the randomness of the sample before and after the creation of subsamples on the basis of that feature. Essentially IG measures the decrease in entropy when a feature is present versus when it is absent. For details see Section B in S1 File. To implement IG we employed the Ranker [41], a heuristic search method that ranks the features based on their individual evaluations. The features selected were all those that had an IG value greater than 0.

### 3.4 Machine learning classification

A machine learning classification approach was used to predict the period to which each letter belonged, based on the features selected in the feature selection step. The corresponding values of the selected features each of the letters formed a feature vector.

#### 3.4.1 Representation of letters by vectors

Each letter was represented by a feature vector of the form $\left(\vec{x},y\right)$ where $\vec{x}\in X$ is a feature vector consisting of a sequence of values (*x*_1_, … *x*_*n*_), for each feature and a binary label *y ϵ* {*e*.*g*. *manic*, *healthy*}.

A machine learning classifier is provided as input with a set of vector representations of letters already assigned to a period (training data). The trained classifier is then given a new set of unseen letters (test data), each of which it assigns to one of the groups using the model derived from the training data. Naive Bayes (NB) [42] and Multilayer Perceptron (MLP) [43] were used as machine learning classifiers. More detailed descriptions of the classification procedures, and of the Naïve Bayes and Multilayer Perceptron classifiers are given in Section C in S1 File.

### 3.5 Evaluation procedures

To evaluate the feature selection and classification procedures we adopted a five-fold cross validation approach. First, the letters used for the classification task were randomly divided into five equally sized, randomly selected subsets, four of which were used to select the features in the feature selection stage, and the same four to train the classification algorithm. The remaining subset was used to test the classification algorithm based on the selected features from the four training subsets. The feature selection and classification steps were repeated five times, with a different subset used for testing purposes each time, and training on the remaining four subsets.

### 3.6 Stability analysis

To evaluate the temporal stability of the written language characteristics during manic and healthy periods over time, letters written between October 1770 to April 1771 (a mentally healthy period of the king’s life) and those written during the period of acute mania were subdivided into three equally sized, sequential subsets. To look for variation in the values associated with each of the 29 features derived from these letter sets across the two periods in question, values from each subset were compared using a series of one-way ANOVAs.

## 4. Results

### 4.1 Feature selection

The majority of selected features were almost identical in all iterations of each feature selection task. Table 3 displays the features that exhibited the greatest overlap among all the five iterations for each feature selection task listed are ranked in decreasing IG order. The IG value for each feature is the average value from the five iterations. The contribution of each selected feature is further illustrated by a comparison of the mean values of the features selected in each contrast.

**Table 3:** Feature selection results for contrasts A to F.

Contrast			Features selected	IG value	Average feature value in each text set	
	1	2			1	2
A	Pre-mania	Acute mania	DL/V	0.35	0.12	0.09
			W	0.33	9.55	8.56
			Log TTR	0.17	0.91	0.92
B	Acute mania	Post mania	W	0.34	9.62	8.68
			CR	0.3	0.59	0.64
			Log TTR	0.21	0.91	0.94
			H	0.2	0.95	0.96
			ADVV	0.1	0.037	0.05
C	Acute mania	Mentally healthy, no stressors	W	0.34	9.61	8.74
			Log TTR	0.21	0.91	0.93
			H	0.15	0.95	0.96
			CR	0.15	0.59	0.64
			LV	0.15	0.89	0.93
			CP/T	0.12	0.62	0.39
D	Acute mania	Mentally healthy, political stressors	C/S	0.28	4.19	5.95
			MLS	0.27	44.01	55.32
			CP/T	0.18	0.62	0.37
			CP/C	0.14	0.2	0.12
E	Mentally healthy: Spring & Summer	Mentally healthy: Autumn & Winter	None			
F	Mentally healthy: Autumn & Winter	Mentally healthy: Spring & Summer	None			

In comparison A (Acute Mania vs Pre Mania) the comparative mean values of all the selected features shown in Table 3 imply the emergence of a less varied vocabulary during the manic period: Dis legomena over Vocabulary (i.e. words that are repeated twice divided by the total number of unique words in the transcript), and those that describe vocabulary richness (Brunet’s ‘W’; MS Log TTR) were the most highly informative.

In comparison B (Acute Mania vs Post-Mania), the selected features were those that described vocabulary richness (Brunet’s ‘W’; MS Log TTR), information content (H and CR), and lexical variation (ADDVV), indicating a poorer, more redundant, patterned, predictable, and repetitive use of language during the mentally unwell period. Letters written after the end of the manic phase were associated with higher mean variation in adverb usage than those of the manic phase, consistent with greater lexical diversity following the illness.

Features selected in comparison C (Acute Mania vs. 1770–1771) were also relevant to vocabulary richness (Brunet’s ‘W’; log TTR), predictability and redundancy (H and CR), lexical variation (LV) and syntactic complexity (CP/T). Comparisons of the mean values associated with the two data sets followed those of comparison B insofar as they indicated a less lexically variable, more repetitive and less informative use of language associated with the manic phase. In accordance with these results, and with respect to the Lexical Variation (LV) measure we found that the diversity of lexical or content words used during the manic phase was also significantly restricted, compared to the healthy period.

By contrast, the values of the syntactic features reveal a significantly higher rate of coordinate phrases (i.e. a verb, verb phrase, adjective phrase, adverb phrase or noun phrase dominating a coordinating conjunction, e.g. ‘and’, ‘but’, ‘for’, ‘or’, ‘nor’, ‘yet’, and ‘so’) in the manic period. This extensive use of phrase coordination indicates a tendency to connect consecutive propositions in a seamless flow, mirroring the rapid, ‘pressured’ speech (rapid talking, which is often loud and can be difficult to interrupt and may be difficult to understand) with frequent topic shifts (‘flight of ideas’) characteristic of spoken language behaviour during a manic episode [44].

In comparison D (Acute Mania vs. 1780–1781) all the selected features were relevant to syntactic complexity. Specifically, while the number of clauses per sentence and the sentence length were both higher in the healthy than the manic phase, the use of coordinate phrases was again higher, both per T-unit (CP/T) and per clause (CP/C) in letters dating from the manic phase. The existence of longer sentences during the healthy phase is, therefore, not regarded as deriving from an increased use of complex phrases, such as coordinate phrases, but from an increased number of clauses. These range from structures with a subject and a finite verb to independent clauses, adjectival, adverbial and nominal clauses. By contrast, increased complexity at the clausal level during the healthy phase could indicate greater eloquence, as the correct use of subordinate clauses is associated with more advanced writing styles, while the higher phrasal density during the manic phase entails less literary sophistication.

In comparisons E (October 1770 –April 1771 vs April 1770 –September 1770) and F (October 1770 –April 1771 vs May 1771 –October 1771) none of the 29 features used was selected by IG indicating that none was regarded as potentially distinctive in the comparisons in question. This suggests that the periods in question have similar syntactic characteristics, lexical variation, redundancy and informativeness and, therefore, that the language variations that emerged from comparisons involving the manic phase were unlikely to have an explanation based purely on seasonal changes in language use.

### 4.2 Classification

The Naive Bayes (NB) classifier was used for comparisons A to F. In the case of comparisons E and F, the available data sets were highly unbalanced between the two classes and we therefore employed Multilayer Perceptron (MLP) as an additional classifier, as it has been found to be suitable for the classification of unbalanced datasets [45]. For the sake of consistency and completeness Multilayer Perceptron was also employed for comparisons a) through d). Results for the latter comparisons are presented in Table A in S1 File. For comparisons A to D, NB and MLP used the features selected by the IG algorithm while for the comparisons E and F the full set of 29 features was used, as IG did not find any distinct features.

Table 4 shows the confusion matrices (which report the numbers of letters in each comparison that have been classified as True Positive (TP), False Positive (FP), True Negative (TN) and False Negative (FN)) and the micro-average classification accuracy scores, for comparisons A to F. Micro-average accuracy is calculated using the TP, TN, FP and FN counts from each of the five folds, according to Eq 1:

$$\frac{\sum_{i=1}^{n}TP_{i}+TN_{i}}{\sum_{i=1}^{n}TP_{i}+TN_{i}+FP_{i}+FN_{i}}$$ (1)

**Table 4:** Micro-average classification accuracy of ML classifiers bs. Baseline for comparisons A to F.

Comparison		ML classifier^1^			Baseline			
		Correct	Incorrect	Accuracy	Correct	Incorrect	Accuracy	p-value^2^
A	Pre-mania	11	10	0.75	0	21	0.6	< 0.05
	Acute mania	28	3		31	0		
B	Acute mania	26	5	0.76	0	31	0.54	< 0.05
	Post-mania	26	11		37	0		
C	Acute mania	18	13	0.7	0	31	0.6	< 0.05
	Mentally healthy, no stressors	37	10		47	0		
D	Acute mania	25	6	0.69	0	31	0.57	< 0.01
	Mentally healthy, political stressors	26	16		42	0		
E	Mentally healthy: Spring & Summer	38	9	0.72	47	0	0.7	> 0.05
	Mentally Healthy: Autumn & Winter	10	10		0	20		
F	Mentally healthy: Autumn & Winter	34	13	0.62	47	0	0.81	> 0.05
	Mentally healthy: Spring & Summer	2	9		0	11		

^1^ Naïve Bayes (NB) for comparisons A to D; Multilayer perceptron (MLP) for comparisons E & F

^2^ Student's t-tests, classifier vs. baseline

The baseline condition was implemented using the ZeroR classifier (http://chem-eng.utoronto.ca/~datamining/dmc/zeror.htm) provided by WEKA, which simply predicts the majority category. Since it lacks predictability power, ZeroR is only useful for determining a baseline performance as a benchmark for other classification methods. Paired t-tests were conducted on the micro-average accuracies compared to the baseline condition, and were computed separately for each fold.

In the classification of letters from the Acute Mania vs Pre-Mania, and Acute Mania vs Post-Mania periods (comparisons A and B), NB significantly outperformed the baseline condition (p<0.05), indicating that letters written before and after the onset of the manic episode were distinguishable from the letters written during the period of mania on the basis of the selected features.

Table 4 also shows the results for classification tasks C and D. In both tasks NB outperformed the baseline condition (p<0.05), implying that the language of the manic episode was distinguishable from the apparently mentally healthy but psychologically stressful periods associated with political turbulence. Therefore, both comparisons support the existence of an idiosyncratic language pattern associated with the King’s first manic episode.

In classification tasks E and F, MLP failed to significantly outperform the baseline condition (p > 0.05), suggesting that the letters written during a mentally healthy period but with the same seasonal distribution as those from the manic vs. pre- and post-illness periods were indistinguishable. The failure to discriminate between letters written during these three periods supports the hypothesis that the differences characterizing letters written during the manic phase stem from linguistic changes related to the mental disorder rather than being either chance findings or related to other influences.

### 4.3 Stability analysis

The results of one–way ANOVAs comparing the values of each of the 29 features across early middle and late epochs of manic and healthy periods of the king’s life are displayed in Tables 5 and 6. Where the assumption of sphericity was violated a Greenhouse-Geisser correction was used. Applying Bonferroni’s correction for multiple comparisons there were no significant differences in any feature value across the three epochs of the manic or mentally healthy periods, suggesting that throughout both of these periods language characteristics remained stable.

**Table 5:** Stability analysis for letters written during the acute mania period.

Feature category	Feature		MEAN (sd) VALUE IN:							
			Early manic phase		Mid manic phase		Late manic phase		p value^1^	Significance^2^
*Syntactic complexity*	1	MLC	11	(1.38)	11.59	(1.19)	12.2	(3.91)	0.07	ns
	2	MLS	41.49	(13.14)	43.49	(15.25)	47.28	(13.59)	0.73	ns
	3	MLT	28.48	(7.73)	35.47	(10.67)	37.7	(16.53)	0.27	ns
	4	C/S	4.62	(1.27)	3.8	(1.42)	4.1	(1.35)	0.54	ns
	5	C/T	3.13	(0.6)	3.12	(1.1)	3.07	(0.86)	0.99	ns
	6	CT/T	0.85	(0.11)	0.76	(0.24)	0.85	(0.21)	0.5	ns
	7	DC/C	0.59	(0.13)	0.6	(0.14)	0.58	(0.16)	0.93	ns
	8	DC/T	1.9	(0.7)	1.99	(1.11)	1.87	(0.98)	0.94	ns
	9	CP/C	0.19		0.25	(0.16)	0.15	(0.11)	0.18	ns
	10	CP/T	0.61	(0.33)	0.82	(0.68)	0.44	(0.36)	0.25	ns
	11	T/S	1.49	(0.35)	1.24	(0.36)	1.36	(0.46)	0.34	ns
	12	CN/C	1.47	(0.25)	1.51	(0.4)	1.57	(0.76)	0.45	ns
	13	CN/T	4	(1.13)	4.59	(1.54)	4.9	(2.87)	0.07	ns
	14	VP/T	4.02	(0.84)	3.99	(1.85)	4.59	(1.96)	0.65	ns
*Textual*	15	LV	0.88	(0.04)	0.91	(0.04)	0.89	(0.05)	0.88	ns
	16	LogTTR	0.79	(0.02)	0.8	(0.05)	0.77	(0.07)	0.09	ns
	17	NV	0.88	(0.06)	0.94	(0.06)	0.87	(0.08)	0.94	ns
	18	ADJV	0.15	(0.04)	0.13	(0.06)	0.11	(0.05)	0.34	ns
	19	MODV	0.2	(0.03)	0.16	(0.08)	0.14	(0.06)	0.24	ns
	20	ADVV	0.05	(0.02)	0.03	(0.03)	0.04	(0.03)	0.17	ns
	21	CVV	2.85	(0.06)	2.34	(0.69)	2.26	(0.53)	0.08	ns
	22	VV	0.27	(0.05)	0.24	(0.07)	0.24	(0.05)	0.34	ns
	23	W	10.27	(0.5)	9.23	(1.43)	9.23	(0.67)	0.07	ns
	24	HL	9.4	(0.18)	9.32	(0.59)	8.99	(0.56)	0.25	ns
	25	PHL	9.98	(0.04)	9.89	(0.31)	9.99	(0.03)	0.36	ns
	26	D	0.89	(0.02)	0.89	(0.01)	0.89	(0.01)	0.24	ns
	27	DL/V	0.12	(0.04)	0.11	(0.04)	0.13	(0.05)	0.71	ns
	28	H	0.96	(0.01)	0.96	(0.02)	0.95	(0.01)	0.15	ns
	29	CR	0.58	(0.03)	0.63	(0.11)	0.6	(0.04)	0.08	ns

^1^One-way ANOVA

^2^Based on a Bonferroni-corrected threshold of 0.001.

**Table 6:** Stability analysis for letters written during the healthy period October 1770- April 1771.

Feature category	Feature		MEAN (sd) VALUE IN:							
			Early healthy phase		Mid healthy phase		Late healthy phase		p-value^1^	Significance^2^
*Syntactic complexity*	1	MLC	10.02	2.02	10.75	3.98	11.13	3.32	0.38	ns
	2	MLS	44.38	16.28	40.88	11.71	46.04	15.6	0.78	ns
	3	MLT	33.11	13.37	31.84	14.35	27.16	8.22	0.19	ns
	4	C/S	4.45	1.45	4.09	1.43	4.26	1.31	0.9	ns
	5	C/T	3.26	0.99	3.1	1.29	2.56	0.81	0.08	ns
	6	CT/T	0.83	0.23	0.82	0.25	0.82	0.24	0.89	ns
	7	DC/C	0.64	0.12	0.66	0.14	0.58	0.1	0.1	ns
	8	DC/T	2.15	0.95	2.16	1.21	1.47	0.7	0.22	ns
	9	CP/C	0.18	0.15	0.16	0.21	0.08	0.1	0.17	ns
	10	CP/T	0.51	0.38	0.45	0.61	0.22	0.24	0.12	ns
	11	T/S	1.44	0.46	1.4	0.46	1.73	0.5	0.72	ns
	12	CN/C	1.07	0.33	1.17	0.53	1.42	0.46	0.08	ns
	13	CN/T	3.59	1.79	3.7	2.47	3.44	1.18	0.59	ns
	14	VP/T	4.47	1.25	4.15	1.61	3.95	1.13	0.51	ns
*Textual*	15	LV	0.94	0.05	0.92	0.08	0.94	0.06	0.86	ns
	16	LogTTR	0.79	0.03	0.8	0.05	0.798	0.04	0.58	ns
	17	NV	0.93	0.07	0.9	0.1	0.92	0.09	0.69	ns
	18	ADJV	0.1	0.05	0.13	0.06	0.14	0.07	0.22	ns
	19	MODV	0.14	0.07	0.17	0.07	0.18	0.09	0.39	ns
	20	ADVV	0.04	0.04	0.04	0.03	0.04	0.03	0.92	ns
	21	CVV	2.2	0.48	1.79	0.55	2.15	0.59	0.13	ns
	22	VV	0.26	0.04	0.23	0.06	0.28	0.07	0.18	ns
	23	W	8.93	0.75	8.52	0.91	8.78	0.99	0.37	ns
	24	HL	9.03	0.72	9.43	0.56	9.27	0.33	0.2	ns
	25	PHL	9.96	0.17	9.97	0.09	10	0	0.55	ns
	26	D	0.89	0.01	0.89	0.01	0.89	0	0.24	ns
	27	DL/V	0.11	0.04	0.08	0.03	0.08	0.04	0.11	ns
	28	H	0.96	0	0.97	0	0.96	0	0.28	ns
	29	CR	0.63	0.04	0.65	0.06	0.63	0.06	0.4	ns

^1^One-way ANOVA

^2^Based on a Bonferroni-corrected threshold of 0.001

## 5. Discussion

In this study we conducted a series of machine-learning experiments to analyse the texts of letters written by King George III to various correspondents during distinct periods of his life. We used a classification approach to show not only the differences between documents originating from these periods, but also the patterns that support the inference of a period on the basis of a document. As all features were assumed at the outset to be of equal importance), the classification method also revealed which specific features carried the greatest weight in the classification, and therefore which features of the language were predictive of mania.

Letters written during a well-described episode of mental derangement that lasted from October 1788 to April 1789 were compared with control texts, written during periods of mental stability. The mentally stable (‘baseline’) periods considered were: i) the months that immediately preceded and immediately followed the first manic episode; and ii) mentally healthy phases spanning seasonally equivalent ranges of months. Experimental results supported the idea that differences between mentally disordered and healthy periods were detectable in written texts, and that these differences consisted in a number of key linguistic and information theory features.

To examine the possibility that the differences observed may not have been specific to mental illness, we used the same techniques to compare letters written: i) during periods characterised by contrasting levels of political uncertainty; and ii) during periods that spanned the same ranges of months as those used in the mental illness contrast. These two ‘control’ comparisons yielded no evidence of language differences associated with the mental stress associated with governing in politically turbulent times or with seasonal changes.

Finally, to eliminate the possibility that the lexical and syntactic differences between the mentally disturbed and mentally healthy phases reflected a uniform trend rather than large, random deviations from an otherwise stable baseline, we divided these periods into three consecutive sub-epochs of equal length and looked for variations in the mean values of all the linguistic features studied across the time span. None of the features showed any significant degree of variation in either of the two periods studied, implying that the samples, while clearly distinct from each other, were lexically and syntactically homogeneous with respect to the measures used

We naturally expected differences between letters due to factors such as content, style, format, topic or genre to be reflected in the language measures used. For this reason, letters were selected on the basis of their political import: although the king wrote regularly to members of his family, only letters that were addressed to leading politicians of the time, the majority of which contained requests from the king to his ministers about active political issues, were included in the analysis. In this way, the letters’ content was made as homogeneous as possible, and it should also be noted that letters belonging to this ‘political genre’ followed a homogenous, even formulaic style, illustrated in the texts reproduced in the Supplementary material.

Whilst classification using the Naive Bayes and Multilayer Perceptron ML algorithms provided evidence of a principled and measurable difference between letters written under conditions of mental illness and wellbeing, insights into the linguistic nature of these differences were provided by the feature selection stage of the analysis. During the period of mental ill health the king’s letters showed a reduced vocabulary, with fewer distinct word types, but also a tendency to greater redundancy and predictability compared to the letters written prior to onset or after recovery. The subset of results that were relevant to the syntactic complexity of the king’s letters provided evidence of additional impairment at this level.

Among the measures derived from information theory were lower entropy and a higher compression ratio in the texts of letters written during mania than in those from healthy baseline periods. This finding was in keeping with the intuition that a less entropic text implies more ordered and predictable use of language, incorporating a greater degree of repetitiveness and redundancy [37, 46]. The approach also suggests a role for information theory measures in quantifying the effects on language of bipolar disorder and other forms of psychiatric morbidity [11] in clinical populations of patients suffering from these conditions.

It is from these feature selection results that we may potentially derive objective clues to the true clinical diagnosis of King George’s recurrent episodes of mental derangement. As outlined in the Introduction, evidence for the once popular Porphyria theory has been thoroughly discredited [9], and in the modern classification of mental illness acute mania now appears to be the diagnosis that fits best with the available behavioural data [17].

The results of this study provide a timely opportunity to ask whether the features identified as important to ML classification overlap with any recognised characteristics of discourse produced by patients with a diagnosis of acute mania. To our knowledge there are no large-scale evaluations of written discourse in this patient group, so a simple comparison is not (yet) possible. Studies of *spoken* output in patients in the manic phase of bipolar disorder, however, have documented a reduced lexical variety, which may be related either to a repetitive quality of speech [44], or to a concomitant impairment of verbal memory [46]. The reduction in syntactic complexity has been shown to be characteristic of discourse in schizophrenia [47] but we know of no reports of a similar deficit in patients with mania. A number of studies suggest that there is no single, homogeneous pattern of linguistic phenomena associated with mania [48]: those in which mania was contrasted with the depressed phases of bipolar disorder [49, 50] found no differences in lexical richness or in sentential syntactic complexity between the two groups. The former additionally reported more ‘state of being’ verbs (be, being, been), adverbs, first-person and personal pronouns, as well as greater concreteness in the output of manic patients. None of these differences was identified in the present study.

### 5.1 Future work

These gaps and discrepancies in the literature prevent us from claiming that our own findings provide additional, independent evidence for a diagnosis of acute mania as the cause of King George’s mental derangement. We would, however, argue that our study indicates the importance and the nature of the further work that will be required to test this hypothesis. In terms of importance, we contend that the ability of an unbiased ML classifier to identify differences between texts written under conditions of mental health vs. mental illness (but not, crucially, between those written under conditions of mental stress vs. mental tranquility, or of one part of the year vs. another) indicate that there is an underlying set of regularities that is susceptible to further analysis. Furthermore, a description of this ‘clinical phenotype’ may allow us, and others, to look for evidence of similar periods of mental instability—both documented and undocumented—during other periods of King George III’s reign, and in other historically significant figures whose written output has been extensively archived.

As to the nature of the required data, the most pressing need is to acquire a set of prospectively collected texts composed by a contemporary series of patients with diagnoses of acute mania, with a view to examining the impact of this mental state on measures of syntactic complexity and Shannon entropy. Our methodological approach should also be broadened with a view to detecting the lexical changes reported in [50]–namely, state of being verbs, modifying adverbs, first-person pronouns, and personal pronouns. We should also be able to enrich our feature set with word or character n-grams and frequencies of grammatical parts of speech that have been exploited in studies of discourse and authorship [51–53]. Finally, written manuscripts contain an additional and potentially highly informative source of data in the physical structure of the handwriting itself. Our group (the ‘Cognitive Archaeology’ collaboration) will shortly be publishing some preliminary analyses of longitudinal changes in this important domain.

### 5.2 Limitations of the study

Any study that imposes contemporary descriptive categories on historical phenomena is open to criticism. The debate surrounding the appropriateness of using modern medical terminology in historical contexts is well known and need not be rehearsed here. Differences at the linguistic level, however, are relevant to our methodology, recognition of which may bring about incremental improvements in the analysis technology in the future. The first point to note is that contemporary orthography made frequent use of semicolons (as well as the marks listed in the footnotes to Table 2) to mark sentence boundaries, leaving the definition of a sentence used by Lu’s syntactic complexity analyzer in need of modification when applied to written discourse of this era. Moreover, agreed definitions of any linguistic terms (‘clause’, ‘phrase’) may be so elusive as to preclude satisfactory implementation in a rule-based computational model. We would point out, though, that the motivation for this study was pragmatic rather than linguistic: we aimed to describe and test a method for characterising patterns of written language usage that reliably predicted the presence of known mental derangement, and could potentially be used to argue for the presence of similar, undocumented changes both in King George and other important historical figures. Finally, we acknowledge the limitations imposed by the application of: i) a method of complexity measurement that was developed specifically for research into second language acquisition rather than to characterise native users’ responses to psychological influences; ii) tools developed for synchronic language research to characterise diachronic data, where their high levels of accuracy cannot be verified. Comparative analyses of larger samples of diachronic material would help to define a control condition more accurately, but were beyond the scope of the present study. Future refinements of our methods, to address the above shortcomings and improve the accuracy of this novel and potentially powerful historical data mining approach will benefit from the addition of a theoretical linguist to the established interdisciplinary collaboration.

## Supporting information

S1 File Lexical and Syntactic complexity analyzers (Section A). Feature Selection (Section B). Machine learning Classification (Section C). Sample texts from the analysed corpus (Section D). Additional classification results using Multilayer Perceptron (Table A). Multilayer Perceptron with three Layers (Figure A).
(DOCX)
