# Supplementary material for: The acute mania of King George III: A computational linguistic analysis
Source: PLoS One. 2017 Mar 22;12(3):e0171626. doi: 10.1371/journal.pone.0171626 (PMC5362044; doi:10.1371/journal.pone.0171626)
Supplement: S1 File — Lexical and Syntactic complexity analyzers (Section A). Feature Selection (Section B). Machine learning Classification (Section C). Sample texts from the analysed corpus (Section D). Additional classification results using Multilayer Perceptron (Table A). Multilayer Perceptron with three Layers (Figure A). (DOCX) [file pone.0171626.s001.docx]

# Supporting Information (SI)

## Lexical and Syntactic complexity analyzers (Section A)

The Syntactic Complexity analyzer [[1](#_ENREF_1)] employed in the current study invokes the [Stanford parser](http://nlp.stanford.edu/software/lex-parser.shtml) [[2](#_ENREF_2)] to parse the input file and [Tregex](http://nlp.stanford.edu/software/tregex.shtml) [[3](#_ENREF_3)] to query the parse trees.

Stanford parser is trained using native language data from the Penn Treebank therefore it is appropriate for analyzing a native speaker’s language. Stanford parser is also recommended because of its reported high accuracy (F-score of .867) for constituent labeling. All the syntactic complexity variables adopted are operationalized with the assistance of the Tregex patterns [[3](#_ENREF_3)] employed by the Syntactic Complexity Analyzer used. Tregex patterns are applied on the output parse trees created by Stanford Syntactic Parser. The way syntactic complexity variables are operationalized by Tregex patterns is described in Lu, 2010 [[1](#_ENREF_1)].

Moreover the Lexical Complexity analyzer [[4](#_ENREF_4)] uses the Stanford Part of Speech (POS) tagger [[5](#_ENREF_5)], which assigns every token in the language sample a label that indicates its part-of-speech category. Stanford POS tagger is also known for its high accuracy. Lexical complexity analyzer also employs MORPHA [[6](#_ENREF_6)] a robust morphological analyzer for English that returns the lemma and inflection of a word. All the aforementioned details for the tools that both lexical and syntactic complexity analyzers are using can be found in [[1](#_ENREF_1), [4](#_ENREF_4)] respectively.

## Feature Selection (Section B)

Information Gain (IG) measures the decrease in entropy when a feature is present versus when it is absent. This can be formally described as follows:

For a binary classification into groups A and B, entropy $H$ over a sample $\left(T\right)$ is formally defined as:

$$H\left(T\right)=-P\left(A\right)\text{log}\left(P\left(A\right)\right)-P\left(B\right)\text{log}\left(P\left(B\right)\right)$$

Eq. S1.

where $P\left(A\right)$ and $P\left(B\right)$ are the probability density functions for $A$ and $B$. The information gain (IG) for a feature $X$ in a sample $T$ is:

$IG\left(T,X\right)=H\left(T\right)-H\left(T|X\right)$ Eq. S2.

which becomes:

$IG\left(T,X\right)=H\left(T\right)-\sum_{v\in values\left(X\right)} \left(\frac{|T_{v}|}{|T|}\right)H\left(T_{v}\right)$ Eq. S3.

where values $\left(X\right)$ is the set of possible values for feature $X$ and $T_{v}$ is the subset of $T$ in which feature $X$ has value v. A feature whose values all belong to only one of the two categories would have an entropy of 0, giving the feature a very high value of IG, while one whose values always belong to both categories would have an entropy value closer to 1, and a lower IG value.

## Machine learning Classification (Section C)

### Naïve Bayes Classifiers

Naïve Bayes (NB) classifiers are implementations of Bayes’ theorem, which concerns the degree to which the likelihood of a hypothesis being correct is contingent on previously unknown information. The term ‘naive’ originates from the fact that the classifier assumes the features used to classify texts to be conditionally independent given the class. Although the assumption of independence is not always true, NB is said to perform well even on complex tasks where it is clear that the strong independence assumptions are false.

More formally, to calculate the probability of observing a feature vector $\vec{x}$ comprising features $x_{1}$ to $x_{n}$, given a class $c$ under the NB assumption, the following holds:

$p\left(\vec{x}|c\right)=p(x_{1},...,x_{n}|c)=\prod_{i=1}^{m} p(x_{i}|c)$ Eq. S4

In order for NB to be used as a classifier for a new letter $\vec{x}$ it is easier to work with the posterior probability (i.e. that the hypothesis is correct given some new information):

$p\left(c|\vec{x}\right)=p(c|x_{1},...x_{n})\propto p(c)p(x_{1}|c)...p(x_{n}|c)$ Eq. S5

or

$$p(c|\vec{x})\propto p\left(c\right)\cdot p\left(\vec{x}|c\right)$$

where $p\left(c\right)$ refers to the prior probability that a letter belongs to class $c$, which according to the maximum likelihood estimate is simply the ratio of the number of letters belonging to the particular class over the overall number of letters. The prior probability for the class Acute Mania is therefore (31/68) = 0.45, and for the class Post-Mania (37/68) = 0.54.

The NB classifier computes the class of each letter by finding the one that maximizes the value of$p\left(c\right)\cdot p\left(\vec{x}|c\right)$, using the Bayes probabilistic model (Eq.S2), together with the maximum a posteriori (MAP) decision rule. Therefore, NB classifies a letter to a class using the classification function:

$classify\left(\vec{x}\right)=argmax_{c}p\left(c\right)\prod_{i=1}^{m} p\left(x_{i}|c\right)$ Eq.S6

where $c$ is one of the possible classes; $argmax_{c}$ indicates the class with the highest value for the function that follows it; $p\left(c\right)$ is the prior probability assigned to a given class; and $p(x_{i}|c)$ is the probability that the word feature with the value $x_{i}$ belongs to a transcript of class $c$.

NB classifiers have a number of different versions, which calculate $p(x_{i}|c)$in different ways. The version employed was Naive Bayes Gaussian (NB) described in the following paragraph.

### Naive Bayes Gaussian (NB)

The value of the probability $p(\vec{x}|c)$ is obtained under the assumption that the features are normally distributed across the transcripts in the corpus. The class for each letter is therefore computed using the formula:

$p(\vec{x}|c)=\prod_{i=1}^{m} g(x_{i};\mu_{i,c},\sigma_{i,c})$ Eq.S7

where $g(x_{i}$..) is the normal distribution for each feature in each category $c$, $\mu$ is the mean, and $\sigma$is the standard deviation of these distributions. By analogy with equations Eq.S6 and Eq.S7, we obtain the following classification function:

$classify\left(\vec{x}\right)=argmax_{c}p\left(c\right)\prod_{i=1}^{m} g\left(x_{i};\mu_{i,c},\sigma_{i,c}\right)$ Eq. S8

### Multilayer Perceptron

Multilayer perceptron is a development of the Perceptron neural network model, that was originally developed in the early 1960s [[7](#_ENREF_7)]. In machine learning, the perceptron is an algorithm for supervised classification of an input into one of several possible non-binary outputs. It consists of multiple layers of nodes in a directed graph, with each layer fully connected to the next one. Except for the input nodes, each node is a unit (or processing element) with a nonlinear activation function. MLP utilizes a supervised learning algorithm (back-propagation) for training the network.


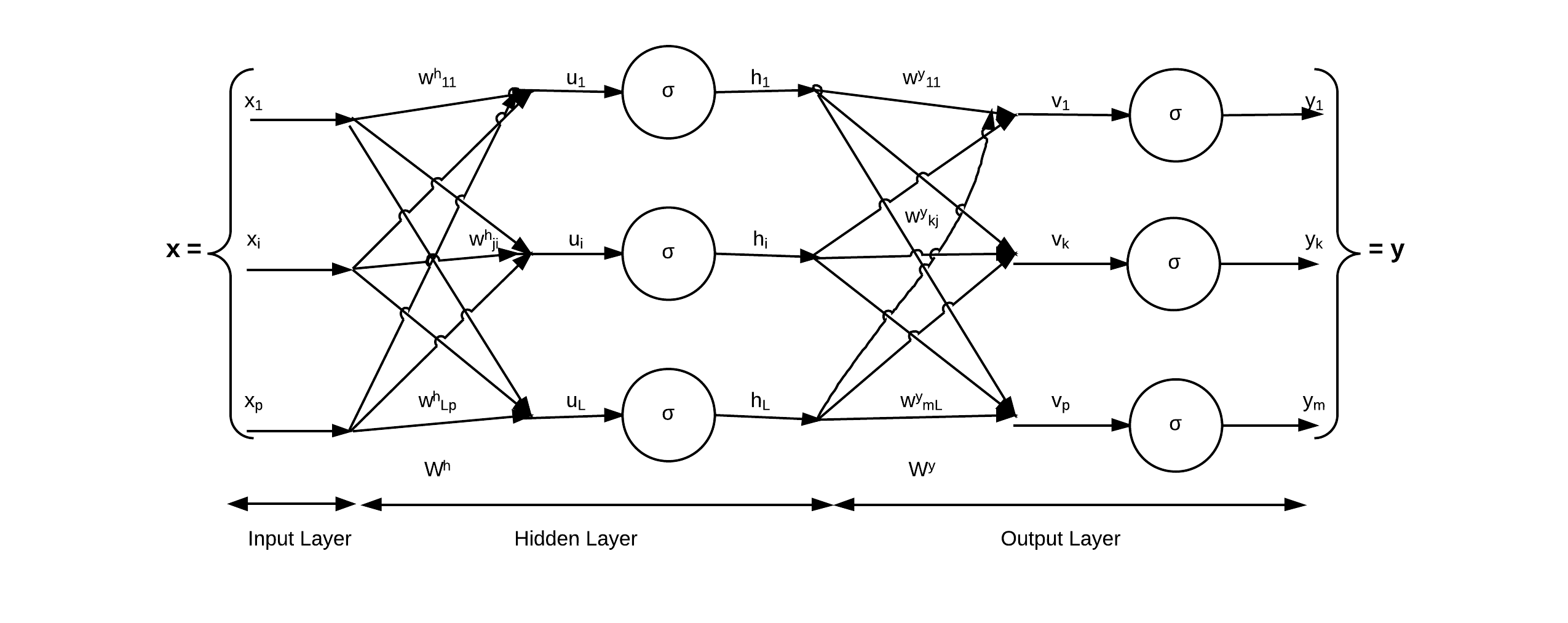


Figure A

The multilayer perceptron employed here consists of three layers (an input and an output layer with one hidden layer) of nonlinearly-activating nodes. The output from each input and hidden node is distributed to all of the nodes in the following layer. Figure S1 illustrates a perceptron network with three layers, and one node in the input layer for each predictor variable. A vector x of predictor (feature) values is presented to the input layer. The values of the features assigned to the input layer together with a constant input of 1.0 (bias) are fed to each of the nodes in the hidden layer, multiplied by a weight, and added to the sum going into the next node. Arriving at a neuron in the hidden layer, the value from each input neuron is multiplied by a weight $\left(w_{ji}\right)$, and the resulting weighted values are added together producing a combined value $u_{i}$. The weighted sum $\left(u_{i}\right)$ is fed into a transfer function, $\sigma$, which outputs a value $h_{i}$. The outputs from the hidden layer are distributed to the output layer. Arriving at a node in the output layer, the value from each hidden layer neuron is multiplied by a weight $\left(w_{kj}\right)$, and the resulting weighted values are summed to produce a combined value $\upsilon_{i}$. The weighted sum $\left(\upsilon_{i}\right)$ is fed into a transfer function,$\sigma$, which outputs a value $y_{k}$.

The weighted sum $\left(\upsilon_{i}\right)$ is fed into a transfer function, σ, which outputs a value $y_{k}$. The $y$ values are the outputs of the network, and give us the output classes for each letter (e.g. manic or healthy).

In the training step we find a globally optimal solution, referring to the values of weights$\left(w_{j}\right)$. If the error is plotted as a function of the weights a ‘rough surface’ with many local minima would result. Several methods have been proposed for eliminating the effects of local minima [[7](#_ENREF_7)], the simplest of which is just to specify a number of random starting points.

A learning rate (the rate at which the weights are updated) of 0.3 was used. The initial weights of the connections between nodes were assigned by a random number generator.

For a classification problem with categorical target variables, there are 2 neurons in the output layer, one for each of the 2 categories of the target variable, and the class with the maximum output was used.

$classify\left(\vec{x}\right)=argmax_{c}y_{c}$ Eq.9

**Table A**: **Additional classification results using Multilayer Perceptron for comparisons A-F**

|  |  | **MLP Classifier** | |  | **Baseline** | |  |  |
| --- | --- | --- | --- | --- | --- | --- | --- | --- |
| **Comparison** |  | **Correct[[1]](#footnote-1)** | **Incorrect[[2]](#footnote-2)** | **Accuracy** | **Correct** | **Incorrect** | **Micro-Average Accuracy** | **p-value[[3]](#footnote-3)** |
| A | Pre-mania | 27 | 4 | 0.73 | 0 | 21 | 0.6 | < 0.03 |
|  | Acute mania | 11 | 10 |  | 31 | 0 |  |  |
| B | Acute mania | 24 | 7 | 0.72 | 0 | 31 | 0.54 | < 0.02 |
|  | Post-mania | 25 | 12 |  | 37 | 0 |  |  |
| C | Acute mania | 16 | 15 | 0.65 | 0 | 31 | 0.6 | < 0.04 |
|  | Mentally healthy, no stressors | 35 | 12 |  | 47 | 0 |  |  |
| D | Acute mania | 20 | 11 | 0.78 | 0 | 31 | 0.57 | < 0.0001 |
|  | Mentally healthy, political stressors | 37 | 5 |  | 42 | 0 |  |  |
| E | Mentally healthy: Spring & Summer | 38 | 9 | 0.72 | 47 | 0 | 0.7 | > 0.05 |
|  | Mentally healthy: Autumn & Winter | 10 | 10 |  | 0 | 20 |  |  |
| F | Mentally healthy: Autumn & Winter | 34 | 13 | 0.62 | 47 | 0 | 0.81 | > 0.05 |
|  | Mentally healthy: Spring & Summer | 2 | 9 |  | 0 | 11 |  |  |

Micro-average classification accuracy of MLP classifier versus the Baseline approach for comparisons A-F.

## Sample Texts from the analysed corpus (Section C)

### Letters derived from the Manic Phase (October 1788 – April 1789) [[8](#_ENREF_8), [9](#_ENREF_9)]

3-11-1788 The King thinks it must give Mr. Pitt pleasure to receive a line from him. This will convince him the King can sign warrants without inconvenience: therefore he desires any that are ready may be sent, and he has no objections to receive any large number, for he shall order the messenger to return to town and shall sign them at his leisure. He attempts reading the despatches daily, but as yet without success; but he eats well, sleeps well, and is not in the least now fatigued with riding, though he cannot yet stand long, and is fatigued if he walks. Having gained so much, the rest will soon follow. Mr. Pitt is desired to be at Kew at two or three o'clock, which ever suits him best.

25-10 -1788 Mr. Pitt really seemed distressed at seeing my bodily stiffness yesterday, which I alone exhibited to stop further lies and any fall of the Stocks. For this kindness I shall desire Sir George Baker (who is to call here previous to my setting out for Windsor this morning) on his return to town to call in Downing Street, that if Mr. Pitt is at leisure he may know exactly how Sir George found me. I am certainly weak and stiff, but no wonder. I am certain air and relaxation are the quickest restoratives. But that nothing may be delayed by my present situation, I authorise Mr. Pitt to acquaint the Cabinet that though I can never think whether Sweden is governed by a corrupt King or a corrupt Senate a subject worthy risking the being drawn into a war, yet that if they wish to hold any language (that is never meant to followed up to these dreadful lengths) which may perhaps tend to keep Sweden in its present situation, I do not object to it. Mr. Pitt is desired by me to acknowledge the receipt of this, and to prevent all political papers being sent to me till I meet him on Wednesday at St. James's.

### Letters derived from the healthy period of King George III (1770-1771) [[10](#_ENREF_10)]

17-1-1771 I am sorry Lord Dartmouth declines the offer that has been made to him, Your shewing him my note was very proper as it contained nothing but my sentiments with regard to him; I wish You could call here previous to seeing Mr Frances or that You would see him so early as to call here still within reasonable hour I mean by that ten this Evening.

By a note I have received from Lord Rochford I know what Mr Frances has to propose to You it is that orders may be immediately sent to Mr Harris to return to Madrid upon which the Spanish Ambassador will communicate his fresh instructions, if this is not complied with he is to threaten War ; I could not help assuring Lord Rochford that I thought this a very absurd proposition for that as the Secretary is recalled we ought to know whether we shall have such terms as we can accept for other ways we shall be tomorrow ordering him to return and in less than two days perhaps a new directing him to come home.

27 - 1- 1781 I am no farther Sorry for the House of Commons not being in a Situation to come with the Address this Day, [than] as it shews the unwillingness of Gentlemen to be Members of the Committees of Elections, which as the Law now stands is certainly an essential part of their Duty; perhaps if the business can go on this Day that [sic] two o’ Clock may be more convenient to the House than three on Monday, as the business will then be less interrupted by the presenting the Address.

# References

1. Lu X. Automatic analysis of syntactic complexity in second language writing. International Journal of Corpus Linguistics. 2010;15(4):474-96.

2. Klein D, Manning CD, editors. Accurate unlexicalized parsing. Proceedings of the 41st Annual Meeting on Association for Computational Linguistics-Volume 1; 2003: Association for Computational Linguistics.

3. Levy R, Andrew G, editors. Tregex and Tsurgeon: tools for querying and manipulating tree data structures. Proceedings of the fifth international conference on Language Resources and Evaluation; 2006: Citeseer.

4. Lu X. The relationship of lexical richness to the quality of ESL learners’ oral narratives. The Modern Language Journal. 2012;96(2):190-208.

5. Toutanova K, Klein D, Manning CD, Singer Y, editors. Feature-rich part-of-speech tagging with a cyclic dependency network. Proceedings of the 2003 Conference of the North American Chapter of the Association for Computational Linguistics on Human Language Technology-Volume 1; 2003: Association for Computational Linguistics.

6. Minnen G, Carroll J, Pearce D. Applied morphological processing of English. Natural Language Engineering. 2001;7(03):207-23.

7. Delashmit WH, Manry M, editors. Enhanced robustness of multilayer perceptron training. Signals, Systems and Computers, 2002 Conference Record of the Thirty-Sixth Asilomar Conference on; 2002: IEEE.

8. Aspinall A. Later Correspondence of George III:v.1. Dec. 1783 to Jan. 1793.-v. 2.1793-1797.-v. 3. Jan. 1798 to Dec. 1801.-v. 4.1802-1807.-v. 5.1808-1810: University Press; 1962.

9. Stanhope EPHS. Life of the Right Honourable William Pitt: With Extracts from His Ms. Papers: J. Murray; 1879.

10. Fortescue JS. (Ed) Correspondence of King George the Third. Vols I- VI. London: Macmillan; 1927-8.

1. True Positive (first row) and True Negative instances [↑](#footnote-ref-1)
2. False Positive (first row) and False Negative instances [↑](#footnote-ref-2)
3. Paired t-tests (p-value < 0,05), classifier vs. baseline [↑](#footnote-ref-3)
